# [F18] FDG-PET/CT for manual or semiautomated GTV delineation of the primary tumor for radiation therapy planning in patients with esophageal cancer: is it useful?

**DOI:** 10.1007/s00066-020-01701-0

**Published:** 2020-10-26

**Authors:** Franziska Walter, Constanze Jell, Barbara Zollner, Claudia Andrae, Sabine Gerum, Harun Ilhan, Claus Belka, Maximilian Niyazi, Falk Roeder

**Affiliations:** 1 Department of Radiation Oncology, University Hospital, Ludwig-Maximilians-University, Munich, Germany; 2 Department of Radiotherapy and Radiation Oncology, Paracelsus Medical University (PMU), Landeskrankenhaus, Salzburg, Austria; 3 Department of Nuclear Oncology, University Hospital, Ludwig-Maximilians-University, Munich, Germany

**Keywords:** Radiotherapy, PERCIST-TLG, Contouring, Gross tumor volume, Interobserver variability

## Abstract

**Background:**

Target volume definition of the primary tumor in esophageal cancer is usually based on computed tomography (CT) supported by endoscopy and/or endoscopic ultrasound and can be difficult given the low soft-tissue contrast of CT resulting in large interobserver variability. We evaluated the value of a dedicated planning [F18] FDG-Positron emission tomography/computer tomography (PET/CT) for harmonization of gross tumor volume (GTV) delineation and the feasibility of semiautomated structures for planning purposes in a large cohort.

**Methods:**

Patients receiving a dedicated planning [F18] FDG-PET/CT (06/2011–03/2016) were included. GTV was delineated on CT and on PET/CT (GTV_CT_ and GTV_PET/CT_, respectively) by three independent radiation oncologists. Interobserver variability was evaluated by comparison of mean GTV and mean tumor lengths, and via Sørensen–Dice coefficients (DSC) for spatial overlap. Semiautomated volumes were constructed based on PET/CT using fixed standardized uptake values (SUV) thresholds (SUV30, 35, and 40) or background- and metabolically corrected PERCIST-TLG and Schaefer algorithms, and compared to manually delineated volumes.

**Results:**

45 cases were evaluated. Mean GTV_CT_ and GTV_PET/CT_ were 59.2/58.0 ml, 65.4/64.1 ml, and 60.4/59.2 ml for observers A–C. No significant difference between CT- and PET/CT-based delineation was found comparing the mean volumes or lengths. Mean Dice coefficients on CT and PET/CT were 0.79/0.77, 0.81/0.78, and 0.8/0.78 for observer pairs AB, AC, and BC, respectively, with no significant differences. Mean GTV volumes delineated semiautomatically with SUV30/SUV35/SUV40/Schaefer’s and PERCIST-TLG threshold were 69.1/23.9/18.8/18.6 and 70.9 ml. The best concordance of a semiautomatically delineated structure with the manually delineated GTV_CT_/GTV_PET/CT_ was observed for PERCIST-TLG.

**Conclusion:**

We were not able to show that the integration of PET/CT for GTV delineation of the primary tumor resulted in reduced interobserver variability. The PERCIST-TLG algorithm seemed most promising compared to other thresholds for further evaluation of semiautomated delineation of esophageal cancer.

## Introduction

Radiation therapy is a cornerstone of the multimodality treatment of locally advanced esophageal cancer [1, 2], either as definitive chemoradiotherapy (CRT) or as preoperative CRT based on randomized controlled trials [3, 4]. While dose and fractionation concepts have remained relatively constant, a considerable shift towards smaller target volumes resulting in less toxicities has taken place over the past decades. For example, the landmark trial RTOG 85–11, which established concurrent CRT as the standard of care for inoperable locally advanced esophageal cancer in the early 90s, had used generous margins for elective nodal irradiation as well as for a tumor bed boost [3]. In contrast, the CROSS trial establishing the role of neoadjuvant CRT more than a decade later introduced much smaller margins including the GTV without any elective nodal irradiation [4]. Similar (smaller) approaches have been proposed recently by the Expert Consensus Contouring guidelines by Wu et al. [5]. Therefore, accurate delineation of gross tumor volume becomes more crucial, especially when combined with the steep dose gradients achieved by modern radiation techniques like intensity-modulated radiation therapy.

CT-based target delineation still represents the standard of care for radiation therapy treatment planning of esophageal cancer patients, although precise definition of the exact tumor boundaries can be difficult, especially in the cranio-caudal direction but also towards directly adjacent mediastinal structures. PET/CT offers some general advantages compared to CT as it adds metabolic to anatomic information, thus enabling a more precise differentiation between vital tumor volume and surrounding structures. Therefore, it has been extensively used in diagnosis, staging, and response assessment of cancer patients [6–8]. For radiation therapy planning, PET/CT seems particularly interesting if performed in the treatment position and has been shown to impact target volumes, for example, in lung cancer, head and neck cancer, prostate cancer, and brain tumors [9–12]. While the role of PET/CT in assessment of lymph node involvement of esophageal cancer is well established [13, 14], its role in target delineation of the primary tumor is more controversial. Several studies have reported conflicting results [6, 15–20], which may have been affected by small sample sizes and different methods of PET/CT-based contouring. While most studies used visual interpretation of PET/CT images, others promoted either fixed absolute SUV thresholds, like SUV2.5 or percentages of SUVmax such as SUV20, with varying results [15, 16, 21]. Recently, our group reported even more complex background- and metabolically corrected values such as the PERCIST-TLG algorithm as most promising in a study including solid tumors of different origin [10].

Therefore, the purpose of the current study was to evaluate the value of additional PET/CT information on target delineation of gross tumor volume of the primary tumor in esophageal cancer patients in a larger patient cohort. The first part was designed to answer the question of whether the addition of visually interpreted PET/CT information to the standard CT-based planning approach can result in reduced interobserver variability for manual GTV definition taken as a surrogate for more accurate delineation. The second part should answer the question of whether semiautomatic delineation methods may help in the harmonization of GTV delineation, and which particular method should be preferred.

## Methods

### Patients

We retrospectively identified 57 consecutive patients who had been treated with neoadjuvant or definitive radiation therapy for non-metastatic esophageal cancer at our department with a dedicated PET/CT for treatment planning available from 6/2011 to 3/2016. Of these patients, 12 had to be excluded from the analysis, 6 for low [F18] FDG activity (maximum standardized uptake value, SUV_max_, <4), 2 scans showed artifacts from metallic material in the region of the tumor, and in 4 patients the software was unable to generate a semiautomated structure set. Therefore, 45 patients were included in the current study.

### [F18] FDG-PET/CT

All included patients had received a dedicated planning [F18] FDG-PET/CT in supine treatment positioning prior to clinical treatment. Whole-body [F18] FDG-PET/CT scans were acquired from the base of the skull to the proximal femora (GE Discovery 690, General Electric, Munich, Germany). Patients fasted for at least 6 h before PET/CT images were acquired. Emission scans were initiated after a median uptake time of 60 min (range 46–113 min) following intravenous administration of 20 mg of furosemide, 20 mg of butylscopolamine, and [F18] FDG (mean activity 246 MBq, range: 184–322 MBq). Diagnostic CT images using intravenous contrast agent in portal venous phase were acquired in suitable patients. PET images were reconstructed using ordered subset expectation maximization (OSEM)-based algorithms (VUE point FX).

### Manual gross tumor volume delineation

**Fig. 1:**
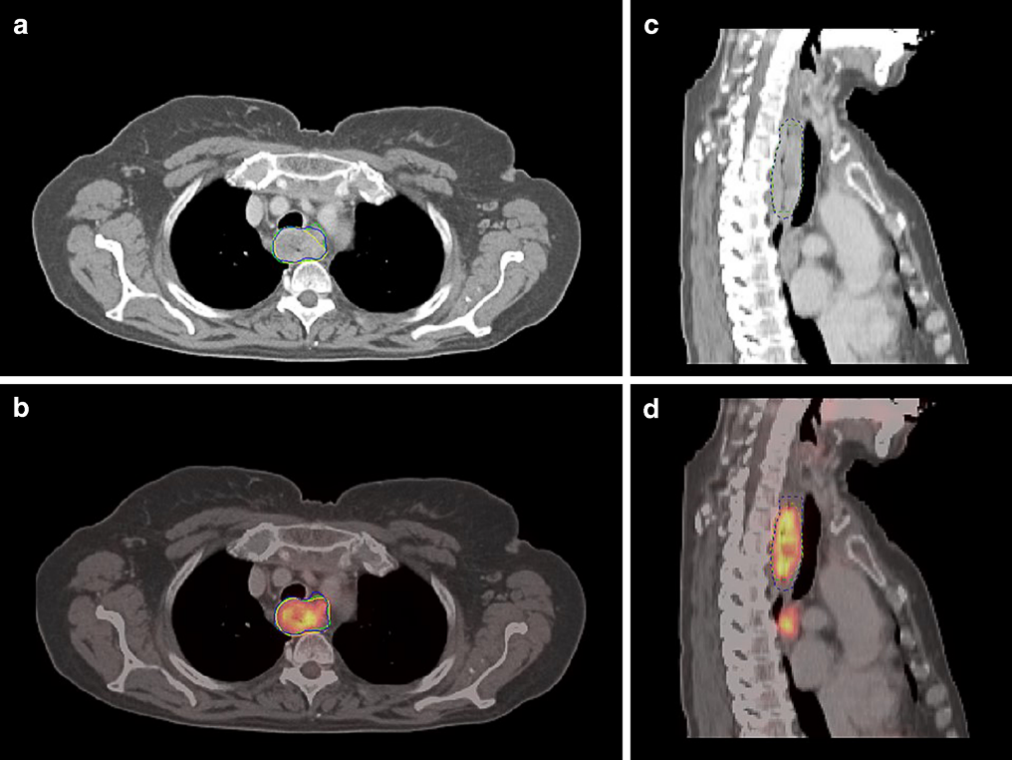
Manual gross tumor volume (GTV) delineation by three observers (*yellow*, *blue*, *green*) **a** on CT alone axial and **b** sagittal; **c** GTV delineation on fused PET/CT axial and **d** sagittal

**Fig. 2:**
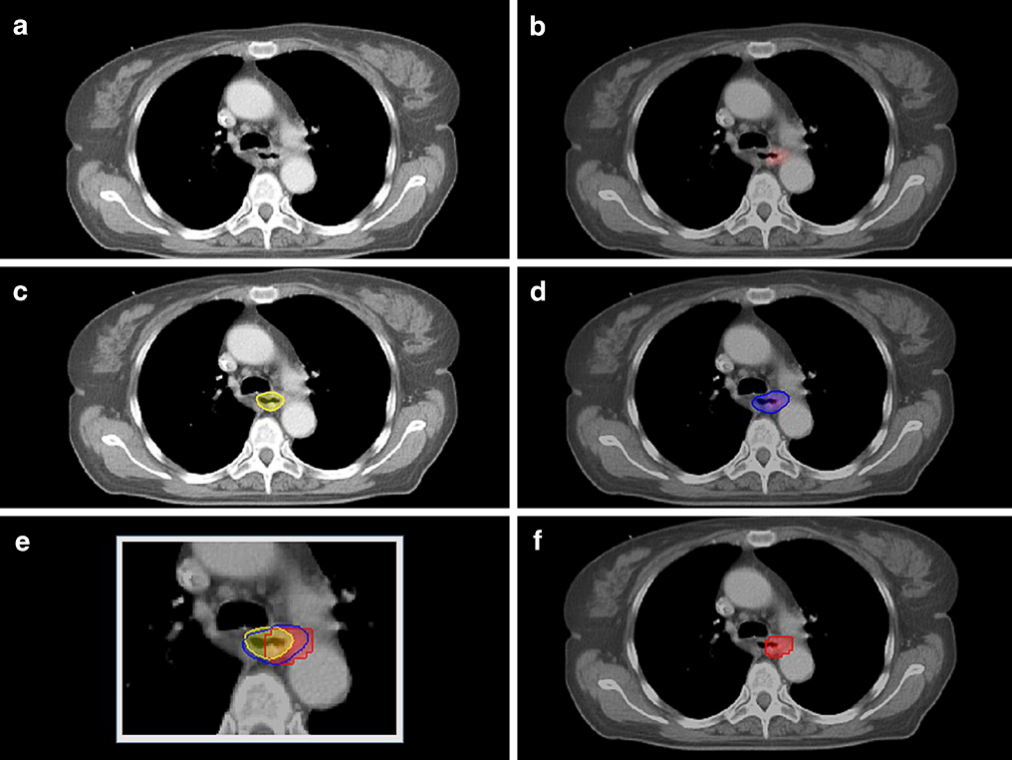
Manual gross tumor volume (GTV) delineation on **a** CT alone, **b** fused [F18] FDG-PET/CT, **c** GTV_CT_ (*yellow*), **d** GTV_PET/CT_ (*blue*), **e** matched GTV_CT_ (*yellow*), GTV_PET/CT_ (*blue*), and PERCIST-TLG (*red*), **f** PERCIST-TLG (*red*) semiautomated contour

Primary tumor delineation was performed by three independent experienced radiation oncologists without prior knowledge of the images or the clinically used target volumes both on PET/CT and CT only. Scans were blinded; only baseline clinical information including TNM stage and tumor extent derived by endoscopy was provided. The radiation oncologists were asked to define the GTV of the primary tumor and include affected lymph nodes only if directly adjacent to the main tumor, since these lymph nodes would also be included in the semiautomatically delineated GTVs. No delineation of nodal volumes (except the above mentioned) or clinical target volumes was performed. GTV of the primary tumor was defined for all studies by each radiation oncologist first on CT scans only (GTV_CT_). To avoid bias in contouring of the PET/CT-based GTV, definition of the respective GTVs on CT including the PET information (GTV_PET/CT_) was done with delay and in random order (Fig. 1). For the manually delineated GTV_PET/CT_, no fixed SUV threshold was provided (visual interpretation; Fig. 2).

### Interobserver variability

**Fig. 3:**
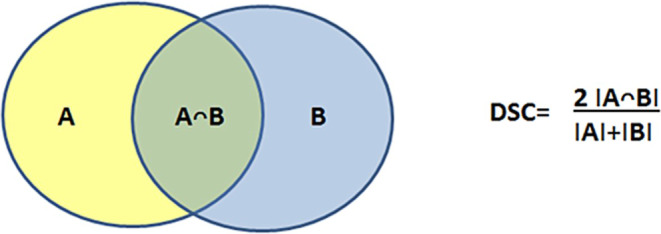
Sørensen–Dice coefficient

Mean tumor volumes and mean tumor lengths of the three observers were calculated per patient for each modality (CT and PET/CT) and compared in non-parametric paired analysis using the Wilcoxon test. To assess geometrical differences, Sørensen–Dice coefficients (Fig. 3) were computed for all three pair of observers and for each modality. Mean dice coefficients of the three observer pairs were calculated for each patient and each modality and compared by the Wilcoxon test.

### Semiautomated gross tumor volume delineation

**Fig. 4:**
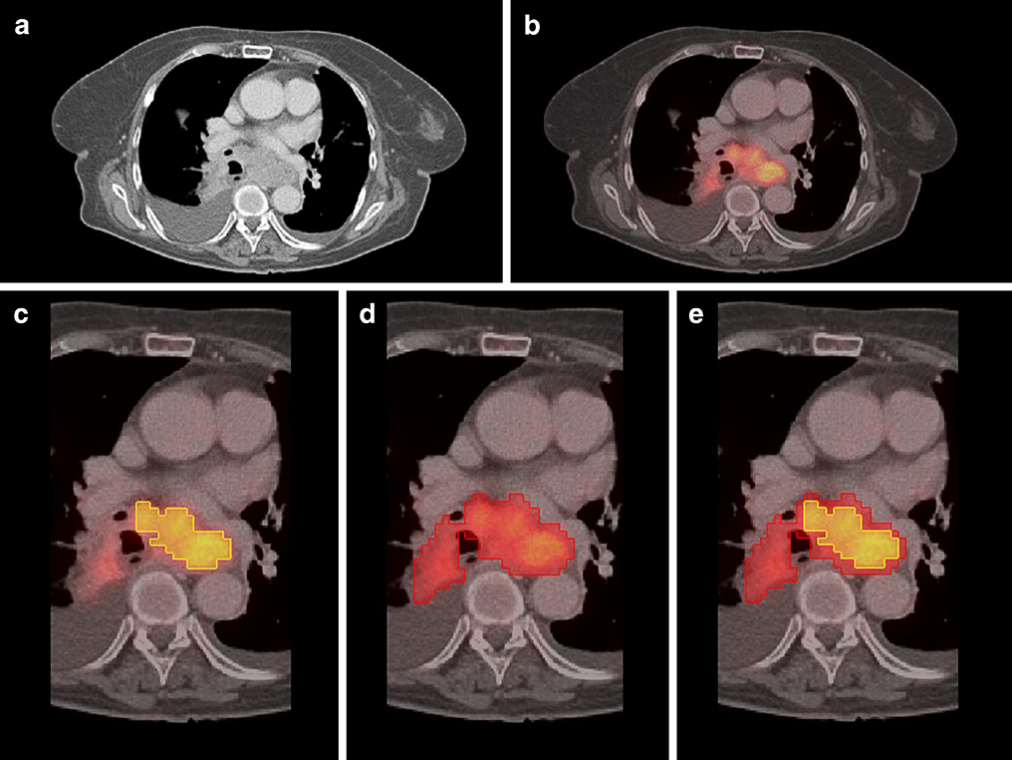
**a** CT alone, **b** fused [F18] FDG-PET/CT, **c** SUV30 semiautomated contour (*yellow*), **d** PERCIST semiautomated contour (*red*), **e** matched SUV30 (*yellow*) and PERCIST (*red*)

Semiautomated GTV delineation based on the given [F18] FDG-PET was performed using a dedicated software package (Hybrid Viewer, Hermes Medical Solutions, Stockholm, Sweden). Semiautomated GTVs were defined for a set of standardized uptake values (SUV) derived from the maximal SUV (SUV_max_): SUV30, SUV35, and SUV40 defined as 30, 35, and 40% of SUV_max_. PERCIST-TLG threshold was determined in analogy to the PERCIST criteria based on normal [F18] FDG background activity in a standardized 15 ml VOI in the right hepatic lobe as described by Niyazi et al. [10]. Schaefer’s threshold was calculated by using the formula TS = *a*xSUV70 + *b*xBG as described by Schaefer et al. ([22]; Fig. 4).

### Concordance of semiautomatically delineated GTVs and manually delineated GTVs

The semiautomatically delineated GTVs of the different methods were compared with the mean manually delineated GTV_CT_ and GTV_PET/CT_ of the three observers by the Wilcoxon test.

Dice coefficients were computed for each patient for the semiautomatically delineated GTVs of every method with the manually delineated GTVs of every modality of the three observers separately. Mean Dice coefficients were then calculated for every observer and compared descriptively between the different methods of semiautomated delineation.

### Statistical analysis

Statistical analysis was performed using the Statistical Package for Social Sciences (SPSS, version 26, SPSS Inc, Chicago, IL, USA). For descriptive analysis of patient characteristics and volumes, mean values and ranges were used. GTVs and tumor lengths were compared using the Wilcoxon test after testing for normality by the Kolgomorov–Smirnov test failed for the majority of parameters.

## Results

A total of 45 [F18] FDG-PET/CT datasets of 45 patients were analyzed. Of these, 39 patients had SCC and 5 had histologically proven adenocarcinoma of the esophagus. Tumors were localized in the cervical (7 patients), upper thoracic (16 patients), middle thoracic (13 patients), and lower thoracic (9 patients) part of the esophagus. Of all patients, 16 had no evidence of lymph node involvement while 29 patients had positive lymph nodes on [F18] FDG-PET/CT. Detailed Patient characteristics are listed in Table 1.

**Table 1:** Patient characteristics

Patient characteristics	
*Gender*	
Male	32
Female	13
*Age*	
Median	69 years
Range	53–85 years
*Histology*	
Adenocarcinoma	5
SCC	39
Neuroendocrine carcinoma	1
*Localization*	
Cervical	7
Upper thoracic	16
Middle thoracic	13
Lower thoracic/GEJ	9
*Grading*	
G1	3
G2	28
G3	14
*T stage*	
cT2	5
cT3	30
cT4	10
*N stage*	
N0	16
N+	29
*RT technique*	
3D-CRT	29
IMRT	16
*RT dose*	
Median	59.4 Gy
Range	12.6–70 Gy
*SUVmax*	
Median	15.6
Range	7.3–51.6

*SCC* squamous cell carcinoma, *GEJ* gastroesophageal junction, *RT* radiation therapy, *SUV* standardized uptake value

### Interobserver variability

Mean primary GTV volumes of all patients based on manual delineation on CT and PET/CT for each observer were 59.2 and 58.0 ml for observer A, 65.4 and 64.1 ml for observer B, and 60.4 and 59.22 ml for observer C. No significant difference between CT- and PET/CT-based delineation was found upon comparing the mean volumes of all three observers per patient on each modality (60.4 ml vs. 59.22 ml, *p* = 0.53). Mean tumor length of all patients based on CT and PET/CT for each observer were 8.1 and 8.1 cm for observer A, 8.3 and 7.6 cm for observer B, and 7.9 and 7.6 cm for observer C. Again, no significant difference between CT- and PET/CT-based delineation was found upon comparing the mean tumor lengths of all three observers per patient on each modality (*p* = 0.11), see Table 2.

**Table 2:** Comparison of manually delineated volumes of different modalities (GTV_CT_ vs. GTV_PET/CT_)

	Three observers		Three observers		Three observer pairs	
Patient	Mean GTV	Mean GTV	Mean length	Mean length	Mean Dice	Mean Dice
Number	CT (ccm)	PET/CT (ccm)	CT (cm)	PET/CT (cm)	CT	PET/CT
1	488.15	488.57	14.83	15.27	0.82	0.86
2	27.97	32.06	5.33	6.07	0.86	0.82
3	182.09	175.51	16.83	16.17	0.89	0.87
4	35.38	38.25	7.03	6.67	0.82	0.87
5	65.04	64.16	8.63	9.20	0.85	0.83
6	41.66	50.73	5.37	5.07	0.79	0.79
7	21.41	18.35	4.03	3.63	0.81	0.81
8	55.57	53.23	9.07	9.70	0.86	0.89
9	25.03	21.96	7.80	5.13	0.86	0.81
10	73.93	57.25	11.40	7.23	0.79	0.81
11	13.98	16.07	4.50	4.20	0.78	0.79
12	24.14	23.00	5.60	5.23	0.65	0.69
13	26.57	27.20	7.30	6.67	0.84	0.81
14	27.72	29.61	6.83	6.53	0.80	0.79
15	7.50	13.82	2.83	4.23	0.76	0.55
16	30.64	31.92	5.60	6.10	0.86	0.88
17	37.98	37.93	7.33	6.57	0.90	0.82
18	17.57	21.20	6.30	8.53	0.76	0.55
19	24.57	28.27	6.90	7.47	0.78	0.77
20	11.87	12.44	5.07	4.43	0.78	0.63
21	39.53	39.84	7.20	6.83	0.82	0.78
22	101.04	128.66	10.83	12.67	0.79	0.80
23	7.50	6.41	4.67	3.00	0.82	0.86
24	40.62	29.49	7.13	5.53	0.77	0.72
25	13.99	16.62	4.57	5.13	0.79	0.74
26	33.47	33.52	7.43	6.97	0.54	0.49
27	11.66	9.87	5.10	4.43	0.48	0.61
28	66.96	69.06	9.17	9.37	0.83	0.79
29	160.32	165.15	10.57	9.17	0.84	0.89
30	37.48	24.96	7.00	4.43	0.86	0.87
31	23.34	18.28	7.33	5.50	0.81	0.68
32	188.17	173.60	12.50	12.17	0.87	0.88
33	137.50	132.84	12.33	10.87	0.85	0.90
34	40.20	33.94	8.77	6.30	0.87	0.84
35	23.65	24.74	3.80	4.13	0.87	0.82
36	30.00	25.19	6.13	4.87	0.87	0.86
37	27.56	26.71	6.00	7.07	0.83	0.74
38	138.39	107.23	21.60	22.33	0.81	0.78
39	65.53	90.83	7.17	6.47	0.76	0.72
40	3.79	7.98	3.30	4.00	0.64	0.67
41	55.96	54.61	7.53	7.80	0.88	0.86
42	81.89	53.03	11.90	10.57	0.68	0.84
43	57.95	54.82	9.80	11.70	0.85	0.81
44	69.44	74.51	9.33	9.47	0.83	0.88
45	22.46	21.53	6.87	6.20	0.85	0.82
Mean	60.38	59.22	7.93	7.58	0.80	0.78
Min	3.79	6.41	2.83	3.00	0.48	0.49
Max	488.15	488.57	21.60	22.33	0.90	0.90

Mean DICE coefficients of all patients on CT and PET/CT were 0.79 and 0.77 for observer pair AB, 0.81 and 0.78 for observer pair AC, and 0.8 and 0.78 for observer pair BC. No significant difference was found between the mean Dice coefficients of all observer pairs per patient between CT- and PET/CT-based delineation (Table 2).

### Concordance of manually and semiautomatically delineated GTVs

The mean SUVmax for the entire cohort was 17.3 (range 7.3–51.5). Mean values for SUV30, SUV35, SUV40, Schaefer’s threshold, and PERCIST-TLG threshold were 5.3 (2.2.–15.5), 6.1 (2.6–18.1), 6.9 (2.9–20.6), 7.0 (3.4–19), and 3.0 (1.4–4.3), respectively. The corresponding mean GTV volumes for SUV30, SUV35, SUV40, Schaefer’s threshold, and PERCIST TLG threshold were 69.1 ml (2.6–22.5), 23.9 ml (1.3–16.0), 18.8 ml (0.7–14.1), 18.6 ml (0.7–12.6), and 70.9 ml (2.3–35.2), respectively.

Comparison of the semiautomatically delineated GTVs with the mean manually delineated GTV_CT_ and GTV_PET/CT_ of all observers resulted in significant differences for all thresholds except for the comparison of PERCIST-TLG threshold with GTV_CT_ or GTV_PET/CT_ (Table 3).

**Table 3:** Volume comparison of manually and semiautomatically delineated GTVsp = 0.826

	Mean GTV_CT_	Mean GTV_PET/CT_
GTV SUV30	*p* < 0.001	*p* < 0.001
GTV SUV35	*p* < 0.001	*p* < 0.001
GTV SUV40	*p* < 0.001	*p* < 0.001
GTV Schaefer	*p* < 0.001	*p* < 0.001
GTV PERCIST-TLG	*p* = 0.722	–

Mean Dice coefficients per observer were calculated for every semiautomatically delineated GTV with both manually delineated GTV_CT_ and GTV_PET/CT_. The best concordance with the manually delineated GTV CT and GTV PET/CT was observed for PERCIST-TLG threshold (mean Dice 0.57–0.6 with GTV CT and 0.61–0.65 for PET/CT; Table 4).

**Table 4:** Mean Dice similarity coefficient comparing manually and semiautomatically delineated GTVs

	GTV_CT_				GTV_PET/CT_			
	Obs. A	Obs. B	Obs. C	Mean	Obs. A	Obs. B	Obs. C	Mean
SUV30	0.54	0.51	0.55	0.53	0.6	0.54	0.61	0.58
SUV35	0.52	0.49	0.53	0.51	0.57	0.51	0.59	0.56
SUV40	0.48	0.44	0.48	0.47	0.51	0.46	0.54	0.5
PERCIST-TLG	0.59	0.57	0.6	0.59	0.64	0.61	0.65	0.63
Schaefer	0.46	0.43	0.46	0.45	0.49	0.44	0.52	0.48

The mean Dice coefficients per observer comparing the manually delineated GTV_CT_ with the GTV_PET/CT_ were 0.77–0.8 (Table 5).

**Table 5:** Mean Dice similarity coefficient of manually delineated GTV CT vs. GTV PET/CT per observer

	Observer A	Observer B	Observer C
Mean	0.77	0.78	0.80
Minimum	0.46	0.46	0.42
Maximum	0.91	0.91	0.95

## Discussion

Adequate delineation of the gross tumor volume of the primary tumor is a prerequisite for successful radiation treatment in general [21]. This is particularly important for the use of modern radiation techniques, with a high level of dose conformity (like IMRT or particle therapy) leading to a higher risk of suboptimal tumor coverage [21] in case of inadequate delineation. CT-based delineation incorporating information from other diagnostic modalities like endoscopy or endoscopic ultrasound is currently the standard approach for delineating the GTV of the primary tumor in esophageal cancer. However, the discriminative value of CT regarding tumor volume and surrounding normal esophageal or mediastinal tissue is often limited, as is the ability to relate spatial information from endoscopy or EUS to the planning CT. As most esophageal cancers show increased FDG uptake [21], especially in locally advanced cases which are typically treated with radiation therapy, it seemed reasonable to evaluate the incorporation of PET/CT into target volume delineation. Several groups have therefore investigated PET/CT-based delineation with regard to various endpoints with different methodologies (summarized in Table 6), thus (not surprisingly) reporting conflicting results [6, 15–20].

**Table 6:** Overview on available literature

Reference	Patients	Objective	SUV thresholds	Reference method	Conclusion
Schreurs et al. [15]	28 EC	Concordance indices GTV, CTV, and PTV	None	CT + EUS	No statistically significant difference in concordance indices No impact on observer variation
Vali et al. [16]	22 EC	SUV threshold for GTV delineation	SUV2.0; SUV2.5; SUV3.0; SUV3.5; SUV40%; SUV45%; SUV50%	CT + EUS	SUV2.5 yields the highest conformality index and best approximates the CT-based GTV at the epicenter
Dong et al. [17]	50 SCEC; 50 NSCLC	Influence of uptake heterogeneity on tumor delineation	SUV40%; SUV2.5	CT	Larger GTV delineation difference in tumors with high FDG uptake heterogeneity
Thomas et al. [18]	20 EC	Tumor volume, tumor length, and volume overlap	SUV2.0; SUV2.5; SUV3.0; SUV20%; SUV35%; SUV40%; SUV45%	CT + clips	CT + clips as “gold standard,” no close agreement with CT alone or PET/CT
Nowee et al. [24]	6 EC	Interobserver variation, CI, most cranial/caudal slice	None	CT, clinical data, EUS	Limited impact on observer variation
Jimenez-Jimenez et al. [19]	29 EC	GTVtumor and GTVnode comparison of volume and tumor length	None	CT	No significant difference in volume of GTVtumor but in *GTVnode*
Toya et al. [20]	10 CEC	Interobserver variation	None	ceCT, barium esophagogram, EUS	PET/CT may increase consistency in GTV delineation in patients with CEC

*EC* esophageal cancer, *SCEC* squamous cell esophageal cancer, *CEC* cervical esophageal cancer, *EUS* endoscopic ultrasound, *VR* volume ratio, *CI* conformity index, *DI* degree of inclusion

Direct correlation of imaging information with pathological specimens is the gold standard to test the validity of an imaging method and has also been studied in esophageal cancer [24–26]. However, this approach requires upfront surgery, which is not the preferred treatment option, especially for locally advanced esophageal cancer in the era of neoadjuvant chemo- or chemoradiation. Analysis of intra- and interobserver variability may serve as a surrogate based on the assumption that lower variability represents more accurate delineation [21].

Therefore, the first aim of our study was to evaluate if the addition of PET/CT to the standard approach based on CT combined with information from endoscopy reduces the interobserver variability of GTV definition regarding the primary tumor in a larger cohort than previously reported. We therefore compared the interobserver variability of three independent observers delineating the primary tumor in 45 cases either with CT or PET/CT with regard to volume and length of corresponding GTVs. Surprisingly, we were not able to detect significant differences between the delineated volumes or the tumor lengths between the two methods. Moreover, concordance between the observers measured by DICE coefficients was not significantly different between the methods, with the absolute values even favoring the CT-based approach (mean Dice coefficient 0.8 vs. 0.78 for CT vs. PET/CT based delineation). These results are in line with the findings of several smaller studies using similar approaches. For example, Scheurs et al. [15] evaluated 28 patients comparing CT- and PET/CT-based delineation by three observers using a concordance index and reported no significant differences. Nowee et al. [6] evaluated 6 cases with 20 observers in a nationwide study in the Netherlands using a conformity index and similarly found no significant reduction in interobserver variability between the two modalities. In contrast, Toya et al. [20] described a significantly reduced interobserver variability for PET/CT compared to CT measured by conformality index in their cohort of 10 patients with cervical esophageal cancer delineated by five observers, with significantly smaller GTVs based on PET/CT imaging. Similarly, Vesprini et al. [27] reported a small but significant reduction in intraobserver variability for the PET/CT-based approach in their study of 10 patients with gastroesophageal cancer. In summary, a benefit of using PET/CT for delineation of the primary tumor volume remains questionable based on the available literature, especially given the negative results of the larger studies including ours. These recent findings confirm the results of a systematic review published by Mujis et al. [21] in 2010, which similarly concluded that the (at that time more limited) available data did not provide sufficient evidence that the integration of PET/CT will necessarily improve the accuracy of GTV delineation in patients with esophageal cancer.

However, these findings should not be misinterpreted regarding PET/CT as a useless tool per se in radiation therapy planning for esophageal cancer. Several groups have described the superiority of PET/CT compared to conventional CT in the detection and delineation of affected lymph nodes, which may lead to substantial changes in nodal GTVs and CTVs [13, 19, 28–30], although inconsistent data from imaging studies on the improvement of sensitivity and specificity of PET/CT compared to other staging modalities exist [21, 31]. Recently, a retrospective analysis of 145 patients treated with neoadjuvant or definitive chemoradiation for esophageal cancer compared patients with or without PET/CT staging with regard to outcome [31]. They found a significantly improved locoregional recurrence-free survival in patients staged by PET/CT with a trend even to improved survival probably based on more accurate target delineation and consequently improved treatment efficacy [31]. Moreover, PET/CT clearly improves the detection of distant metastases compared to conventional CT [32, 33] and therefore seems crucial for adequate patient selection for localized therapies like radiation [31, 33].

Some reasons for the lack of improvement by the addition of PET/CT for delineation of the primary GTV might be simply technical issues. Delineated volumes based on PET/CT may vary extensively based on the contouring method using the metabolic information. Two major types of contouring methods exist, either using visual interpretation (with or without source-to-background correction) or different fixed SUV thresholds. Visual interpretation, which was used in most of the cited studies, is highly observer dependent as image representation can be controlled by changing window widths or window levels, resulting in different visible tumor volumes [21]. On the other hand, SUV as a semiquantitative parameter can be affected by many parameters such as patient preparation, scan acquisition, image reconstruction, and data analysis, which may result in considerable differences in SUV outcome even though most of these effects are small [21]. Nevertheless, semiautomated delineation methods based on fixed thresholds may be helpful in the harmonization of GTV volume definition.

The second aim of our study was therefore to evaluate different SUV-dependent methods with regard to their concordance with manually delineated GTV volumes. Different SUV-based thresholds have been analyzed in the past, either based on fixed absolute values like SUV2.5 [16], based on relative SUV values like SUV30 which represents 30% of the SUVmax of the individual patient [16, 18], or even more complex thresholds including corrections for background or metabolic activity [10]. As thresholds based on absolute values might be additionally influenced by individual patient parameters such as body weight and blood glucose levels, we decided to evaluate relative SUV values (SUV30, 35, and 40). Based on our initial experience, we included the background- and metabolically corrected PERCIST-TLG [10] and Schaefer’s algorithm [22]. Our previous work had described especially the PERCIST TLG algorithm as promising in an analysis including 20 patients with solid tumors including 5 suffering from esophageal cancer [10].

As assumed, we observed considerable differences in the GTV volumes derived from semiautomated delineation with different thresholds, which varied distinctly not only between each other but also compared to the manually delineated GTVs based on CT and PET/CT using visual interpretation. Indeed, all thresholds used resulted in significantly different volumes compared to the mean manually delineated GTVs of the three observers both for CT and PET/CT, except the PERCIST-TLG algorithm.

Regarding the calculated Dice coefficients for the different semiautomatically delineated volumes with the manually delineated volumes of the three observers, we found the highest mean Dice coefficients for the PERCIST-TLG algorithm with manually derived CT (mean Dice coefficient 0.59) as well as PET/CT GTVs (mean Dice coefficient 0.63). However, even the best semiautomated volume (PERCIST-TLG) had a lower concordance with both manually derived GTVs (CT vs. PET/CT) than the GTV_PET/CT_ based on visual interpretation with the GTV_CT_ for every observer (mean Dice coefficient 0.79), indicating a still suboptimal result.

One major drawback for the use of semiautomatically delineated volumes is the common practice of radiation oncologists to always include the whole circumference of the esophagus into the GTV even if visible primary tumor is present only at one side of the esophageal wall. In contrast, all software-based algorithms will contour volumes strictly restricted to detected tumor, which sometimes result in GTVs not covering the whole esophageal circumference (as illustrated in Fig. 4). This issue should be addressed in the design of further studies, either by adding a step to automated contouring to always include the whole esophageal structure in the corresponding CT slice if tumor is automatically detected, or in advising the human observers to strictly contour only the visible tumor regardless of the adjacent esophageal structure.

Our study has some limitations: It is of retrospective nature and therefore may not serve as a confirmative but rather as a hypothesis-generating study regarding at least the design of future studies. Due to the high number of included patients, we decided to limit the number of observers to three, which seems to be the possible minimum (although studies with larger numbers of observers provided similar results). As we focused on primary gross tumor volume, no insights into the possible value of PET/CT for lymph node delineation, clinical target volume definition, or detection of distant metastases can be given. Finally, the common practice of radiation oncologists to include the whole circumference of the esophagus into the GTV even if visible tumor (on imaging) is present only at parts of the circumference makes direct comparisons to semiautomatically detected volumes difficult.

## Conclusion

In summary, we were not able to show that the integration of PET/CT for GTV delineation of the primary tumor in esophageal cancer patients resulted in reduced interobserver variability in a large cohort of patients. Moreover, the evaluated semiautomatically delineated GTVs based on fixed SUV thresholds did not correlate well with the manually derived GTV volumes based either on CT or visually interpreted PET/CT. Nevertheless, we found that the most promising algorithms for further evaluation of semiautomatically delineated volumes probably seem to be background- and metabolically corrected algorithms like PERCIST-TLG, which showed the highest concordance with the manually derived GTVs of all evaluated methods. However, our findings should not be misinterpreted regarding the general value of PET/CT for staging and treatment planning in esophageal cancer patients based on the possible advantages for detection of lymph nodes, including definition of clinical target volumes or the detection of distant metastases. Further studies regarding primary GTV definition should account for general differences between human and semiautomated contouring with regard to inclusion of the whole circumference of tumor-bearing slices of the esophageal structure.

## Abbreviations

CRT: Chemoradiotherapy
CT: Computed tomography
DSC: Sørensen–Dice coefficient
GEJ: Cancer of the esophagogastric junction
GTV: Gross tumor volume
GTV_CT_: Gross tumor volume derived by CT
GTV_PET/CT_: Gross tumor volume derived by PET/CT
IMRT: Intensity-modulated radiotherapy
LoD: Length of disease
PET/CT: Positron-emission tomography/computer tomography
PTV: Planning target volume
SPSS: Statistical Package for Social Sciences
SUV: Standardized uptake values
SUV_max_: Maximum standardized uptake value
VMAT: Volumetric modulated arc therapy
